# Handgrip strength, depression, and all-cause mortality in Korean older adults

**DOI:** 10.1186/s12877-019-1140-0

**Published:** 2019-05-03

**Authors:** Soohyun Park, Jinkyung Cho, Donghyun Kim, Youngyun Jin, Inhwan Lee, Haeryun Hong, Hyunsik Kang

**Affiliations:** 1 Department of Sports Science, Korea Institute of Sport Science, Seoul, Republic of Korea; 2 Laboratory of Exercise Physiology and Biochemistry, College of Sport Science, Sungkyunkwan University, 2066 Seobu-Ro, Jangan-Gu, Suwon, 16419 Republic of Korea

**Keywords:** Aging, Physical dysfunction, Mental health, Premature death, Geriatrics

## Abstract

**Background:**

Decreased muscle strength and/or depression with aging are emerging as important public health concerns in both developed and developing countries. This study investigated the effects of low handgrip strength (HGS) and depression on the risk of all-cause mortality in Korean older adults.

**Methods:**

Data from 13,901 Korean adults (57% women) who participated in the 2008 baseline survey and completed the 2011 follow-up assessments were used.

**Results:**

In total, the current findings showed that individuals with depression only and individuals with low HGS plus depression had significantly higher risks of all-cause mortality (hazard ratio (HR) = 1.366, 95% confidence interval (CI) = 1.033–1.807, *p* = 0.029 and HR = 1.961, 95% CI = 1.409–2.736, *p* < 0.001, respectively) even after adjustments for all the measured covariates, compared with individuals with high HGS plus no depression (HR = 1). Gender-stratified analysis showed that men with depression only and men with depression plus low HGS had significantly higher risks of all-cause mortality (HR = 1.376, 95% CI =1.029–1.841, *p* = 0.031 and HR = 1.861, 95% CI = 1.306–2.651, *p* = 0.001, respectively) even after adjustments for all the measured covariates, compared with individuals with no depression plus high HGS (HR = 1). In women, however, the joint effect of depression and low HGS only remained significant at borderline (HR = 2.603, 95% CI = 0.981–6.908, *p* = 0.055) when adjusted for all the confounders.

**Conclusion:**

The current finding suggested that depression and low HGS were significantly and synergistically associated with the increased risk of premature death from all causes in the Korean geriatric population.

## Background

Decreased muscle strength with aging is associated with a number of structural and functional changes that are conducive to increased disability, frailty, and falls [1]. Handgrip strength, a simple bedside tool, was found to be a valid surrogate measure of overall muscular strength [2]. Low handgrip strength predicts all-cause mortality as well as cardiovascular disease (CVD) [3, 4] and cancer mortalities [5]. Yet, low handgrip strength and its relation to the risks of all-cause and cause-specific mortality are modulated by potential confounders, including age [6], body mass index [7], nutritional status [5], physical activity [3], and comorbidities [8].

The geriatric mental health is emerging as an important public health concern in both developed and developing countries [9]. In particular, late-life depression (LLD) is one of the most prevalent psychiatric disorders and is associated with morbidity and mortality [10], although this has not been found consistently. This variability may be influenced by gender since women have been consistently reported to be at higher risk of developing depression, with rates of depression for women 1.5 to 2 times those of men [11]. In the Cooperative Health Research in the Region of Augsburg (KORA)-Age Study involving 1066 older adults (mean age 76 ± 11 SD years), the risk of low HGS for 3-year all-cause mortality tended to be stronger in women than in men [12]. Contradictory, a systematic review and meta-analysis study involving 2 million men and women showed that low HGS was more strongly associated with increased all-cause mortality risk in men than in women [13]. Other studies also reported gender differences in the relationship between muscular strength and mortality [14]. Together, those previous findings suggest that gender as a moderator may play a role in determining the relationship of low handgrip strength (HGS) and depression with the risk of all-cause mortality in older adults. Furthermore, the mediators linking the mental health to negative health outcomes including premature death are not clear.

Like in Western countries, HGS has been used as an important diagnostic and/or prognostic tool to identify older people with functional limitation and/or at increased risk of age-related diseases in Korea [15]. For example, recent population-based studies showed that low HGS was significantly associated with increased risk of new-onset cognitive dysfunction [16], increased 10-year cardiovascular risk [17], and impaired status of health-related quality of life [18]. Using the sixth Korea National Health and Nutrition Examination Survey (KNHANES VI), Lee et al. [19] showed that low HGS was significantly associated with an increased risk of depression in Korean adults aged 18–80 years.

To the best of our knowledge, however, no previous study using a population-based design has been attempted to examine the relationships of HGS and LLD with the risk of all-cause mortality stratified by gender in Korean older adults. Therefore, gaining insight into the associations would contribute to the development of new or improved options for the prevention of premature death associated with low HGS and/or depression. This study aimed to investigate whether or not gender modulates the effects of low HGS and depression at baseline on the risk of 3-year all-cause mortality in Korean adults aged 60 years and older.

## Methods

### Study design and sample (data source)

By using data from the 2008 and 2011 Living Profiles of Older People Survey (LPOPS), which is a national wide 3-year longitudinal survey by the Ministry of Health Welfare and Family in Republic of Korea, we conducted a population-based prospective study to evaluate the relationship of low HGS and depression with all-cause mortality in Korean older adults.

The design of the LPOPS has been described previously [20]. In brief, a total of 14,071 older adults aged > 60 year participated in the 2008 baseline assessment. During the follow-up period, 800 subjects were excluded due to refusal or loss of contact, resulting in 13,901 subjects (43% men and 57% women) who were included in the study analysis. The Institutional Review Board (IRB) of the Keimyung University reviewed and approved the study protocol in accordance with the Declaration of Helsinki. All participants provided written informed consent to participate in the survey.

### Variables

#### Determination of handgrip strength, depression, and all-cause mortality

Detailed description of measurement procedures for exposures (i.e., handgrip strength and depression) and outcome (i.e., all-cause mortality) has been published elsewhere [21, 22]. In brief, HGS was measured using a hand grip dynamometer (TANITA No. 6103, Tokyo, Japan). Individual HGS values were divided into age and sex-specific quartiles and categorized as high HGS (upper 50th percentile) or low HGS (lower 50th percentile). The Korean version of the short form of the geriatric depression scale (SGDS-K) was administered as a screening measure for depression, as described in detail elsewhere [23]. All-cause mortality was defined as number of deaths from all causes, as described in detail elsewhere [21, 22].

#### Determination of confounders

Body mass index was calculated by dividing body weight (kg) by height squared (m^2^). Socio-demographic (i.e., age, gender, education) and health behavioral factors (i.e., alcohol consumption, smoking, and number of comorbidity and medications) were measured as potential confounders, as described in detail elsewhere [21, 22]. In addition, nutritional status was assessed using the nutrition screening initiative checklist [24]. Disability, cognitive function, and physical activity were assessed using the Korean activities of daily living scale (K-ADL) [25], the Korean version of the mini-mental state examination (MMSE-KC) [26], and the International Physical Activity Questionnaire (IPAQ) short form [27], respectively.

### Statistics

Descriptive statistics were presented as means and standard deviations and frequencies and percentages for continuous and categorical variables, respectively. Independent t-test and Chi-square were used to compare mean differences in continuous and categorical variables, respectively. The Cox proportional hazards model was used to estimate the hazard ratio (HR) and 95% confidence interval (95% CI) of handgrip strength and depression for cumulative all-cause mortality. All analyses were performed taking into account complex sampling weights, using SPSS-PC 23.0 (SPSS Inc., Chicago, IL, USA).

## Results

Table 1 represents the descriptive statistics of the participants in the study. In general, women were older (*P* < 0.001) and less educated (*P* < 0.001) and had higher BMI (*P* < 0.001) than men. Men had higher monthly income (*P* < 0.001), higher MMSE-KC score (*P* < 0.001), lower nutritional risk (*P* < 0.001), lower number of comorbidities, and lower SGD-K score (*P* < 0.001) but were more likely to smoke (P < 0.001) and more likely to consume alcohol (P < 0.001) than women.

**Table 1:** Baseline characteristics of study participants

Parameters	Total (*N* = 13,901)	Men (*N* = 5996)	Women (*N* = 7905)	*p* value
Age (years)	69.54 ± 7.06	68.64 ± 6.53	70.23 ± 7.36	< 0.001
BMI (kg/m^2^)	23.83 ± 3.24	23.36 ± 2.92	24.19 ± 3.43	< 0.001
^a^Education, n (%)				
0–3 years	3836 (27.6)	705 (11.8)	3131 (39.6)	< 0.001
4–6 years	4677 (33.6)	1890 (31.5)	2788 (35.3)	
≥ 7 years	5045 (36.3)	3288 (54.8)	1757 (22.2)	
Income (10,000 won)	155.94 ± 185.47	176.07 ± 203.48	140.67 ± 168.96	< 0.001
Marital status, n (%)				
Single/married not living with anyone	2332 (16.8)	357 (6.0)	1975 (25.0)	< 0.001
Married/living with someone	11,569 (83.2)	5639 (94.0)	5930 (75.0)	
Past/current smokers, n (%)	4617 (33.2)	4134 (68.9)	483 (6.1)	< 0.001
^b^Alcohol consumption, n (%)				
No drinking	8734 (62.8)	2506 (41.8)	6229 (78.8)	< 0.001
two times a week (or more)	2394 (17.2)	2018 (33.7)	375 (4.8)	
Nutrition score	3.15 ± 3.18	2.80 ± 2.98	3.42 ± 3.30	< 0.001
Number of comorbidity	1.78 ± 1.50	1.42 ± 1.32	2.05 ± 1.57	< 0.001
K-ADL (score)	7.19 ± 0.98	7.20 ± 1.01	7.18 ± 0.92	0.265
SGDS-K (score)	4.64 ± 4.37	3.92 ± 4.16	5.18 ± 4.44	< 0.001
MMSE-KC (score)	24.22 ± 4.37	25.69 ± 3.52	23.11 ± 4.52	< 0.001
Death, n (%)	696 (5.0)	430 (7.2)	266 (3.4)	< 0.001

*BMI* Body mass index, *K-ADL* Korean Activities of Daily Living, *MMSE-KC* Korean version of mini-mental state examination, *SGDS-K* Korean version of 15-item geriatric depression scale. ^a^Men (*n* = 113) and women (*n* = 229) had no data available. ^b^Men (*n* = 1472) and women (*n* = 1301) had no data available

Table 2 shows mean values of measured parameters according to handgrip strength level. Older men and women with high HGS were younger (*P* = 0.043 and *P* < 0.001, respectively), heavier (*P* < 0.001 and P < 0.001, respectively), more educated (*P *< 0.001 and P < 0.001, respectively), and more frequently to consume alcohol (P < 0.001 and *P* = 0.019, respectively) than older men and women with low HGS. Older men and women with high HGS had higher income (*P* < 0.001 and *P *< 0.001, respectively), higher average MMSE-KC scores (*P* < 0.001 and *P* < 0.001, respectively), lower number of comorbidities (*P* < 0.001 and *P* < 0.001, respectively), and higher physical activity (*P* < 0.001 and *P* < 0.001, respectively) than older men and women with low HGS.

**Table 2:** Comparison of measured parameters according to handgrip strength

	Handgrip strength					
	Men			Women		
	Low	High	P value	Low	High	P value
N	3130	2866		4073	3.832	
Age (years)	68.80 ± 6.88	68.46 ± 6.11	0.043	70.56 ± 7.58	69.88 ± 7.10	< 0.001
BMI (kg/m^2^)	22.90 ± 2.98	23.84 ± 2.78	< 0.001	23.98 ± 3.50	24.38 ± 3.33	< 0.001
^a^Education, n (%)						
0–3 years	462 (14.8)	243 (8.5)		1778 (43.7)	1353 (35.3)	
4–6 years	1026 (32.8)	864 (30.1)	< 0.001	1382 (33.9)	1406 (36.7)	< 0.001
≥ 7 years	1567 (50.1)	1721 (60.0)		786 (19.3)	971 (25.3)	
Income (10,000 won)	164.70 ± 174.56	187.49 ± 215.86	< 0.001	135.80 ± 177.57	144.85 ± 170.48	0.045
Marital status, n (%)						
Single/married not living with anyone	206 (6.6)	151 (5.3)	0.033	1070 (26.3)	906 (23.6)	0.007
Married/living with someone	2924 (93.4)	2715 (94.7)		3003 (73.7)	2927 (76.4)	
Past/current smokers, n (%)	2146 (68.6)	1987 (69.3)	0.539	268 (6.3)	225 (5.9)	0.398
^b^Alcohol consumption, n (%)						
No drinking	1418 (45.3)	1087 (37.9)	< 0.001	3305 (81.2)	2924 (76.3)	0.019
two times a week (or more)	980 (31.3)	1038 (36.2)		176 (4.3)	200 (5.2)	
Nutrition score	3.23 ± 3.20	2.38 ± 2.67	< 0.001	3.82 ± 3.34	3.04 ± 3.23	< 0.001
Number of comorbidities	1.55 ± 1.40	1.29 ± 1.23	< 0.001	2.22 ± 1.59	1.88 ± 1.53	< 0.001
K-ADL (score)	7.34 ± 1.36	7.06 ± 0.50	< 0.001	7.29 ± 1.17	7.07 ± 0.49	< 0.001
SGDS-K (score)	4.75 ± 4.54	3.01 ± 3.49	< 0.001	6.08 ± 4.58	4.25 ± 4.06	< 0.001
MMSE-KC (score)	25.11 ± 3.85	26.39 ± 2.90	< 0.001	22.33 ± 4.81	23.90 ± 4.05	< 0.001
Death, n (%)	309 (9.9)	121 (4.2)	< 0.001	178 (4.4)	88 (2.3)	< 0.001
Absolute Handgrip strength (kg)	26.03 ± 5.90	36.82 ± 4.60	< 0.001	15.48 ± 3.68	23.00 ± 3.70	< 0.001
Relative Handgrip strength (kg/BMI)	2.22 ± 0.56	2.99 ± 0.46	< 0.001	1.24 ± 0.33	1.82 ± 0.36	< 0.001
Physical activity (Mets/week)	1677.9 ± 1885.2	2182.6 ± 2229.9	< 0.001	1127.6 ± 1428.1	1428.9 ± 1730.5	< 0.001

For continuous variables, values are mean; *BMI* Body mass index, *K-ADL* Korean Activities of Daily Living, *MMSE-KC* Korean version of mini-mental state examination, *SGDS-K* Korean version of 15-item geriatric depression scale. ^a^Men with low HGS (*n* = 75) and high HGS (*n* = 38) and women with low HGS (*n* = 127) and high HGS (*n* = 102) had no data available. ^b^Men with low HGS (*n* = 732) and high HGS (*n* = 741) and women with low HGS (*n* = 593) and high HGS (*n* = 708) had no data available

Table 3 shows mean values of measured parameters according to depression status. Depressed older men and women were generally older (*P* < 001 and* P* < 001, respectively) than non-depressed men and women. Depressed older men had lower BMI (*P* = 0.004 and *P* = 0.047, respectively), lower income (*P* < 0.001 and *P* < 0.001, respectively), lower average MMSE-KC score (*P* < 0.001 and *P* < 0.001, respectively), lower handgrip strength (*P* < 0.001 and *P* < 0.001, respectively), lower physical activity (*P* < 0.001 and *P* < 0.001, respectively), higher nutritional risk (*P* < 0.001 and *P *< 0.001, respectively), and higher number of comorbidities (*P* < 0.001 and P < 0.001, respectively) but were less educated (*P* < 0.001 and P < 0.001, respectively) than non-depressed older men and women. Additionally, depressed older men were less frequently to consume alcohol than non-depressed older men (P < 0.001), with no such difference in alcohol consumption observed in women.

**Table 3:** Comparison of measured parameters according to depression status

	Depression status					
	Men			Women		
	Not-depressed	Depressed	P value	Not-depressed	Depressed	P value
N	4816	1180		5664	2241	
Age (years)	68.15 ± 6.24	70.61 ± 7.23	< 0.001	69.33 ± 7.07	72.51 ± 7.57	< 0.001
BMI (kg/m^2^)	23.44 ± 2.84	23.00.03 ± 3.27	0.004	24.20 ± 3.34	24.14 ± 3.64	0.047
^a^Education, n (%)						
0–3 years	471 (9.8)	234 (19.8)	< 0.001	1915 (33.8)	1216 (54.2)	< 0.001
4–6 years	1478 (30.7)	412 (34.9)		2113 (37.3)	674 (30.0)	
≥ 7 years	2787 (57.9)	500 (42.4)		1478 (26.1)	278 (12.4)	
Income (10,000 won)	188.41 ± 206.03	122.72 ± 133.09	< 0.001	155.01 ± 1188.88	102.76 ± 122.40	< 0.001
Marital status, n (%)						
Single/married not living with anyone	226 (4.7)	131 (11.1)	< 0.001	1208 (21.3)	767 (34.2)	< 0.001
Married/living with someone	4590 (95.3)	1050 (88.9)		4456 (78.7)	1474 (65.8)	
Past/current smokers, n (%)	3228 (67.0)	906 (76.8)	< 0.001	250 (4.4)	233 (10.4)	< 0.001
^b^Alcohol consumption, n (%)						
No drinking	1879 (39.0)	627 (53.1)	< 0.001	4396 (77.6)	1833 (81.8)	< 0.001
two times a week (or more)	1691 (35.1)	327 (27.7)		265 (4.7)	110 (4.9)	
Nutrition score	2.25 ± 2.54	5.21 ± 3.54	< 0.001	2.60 ± 2.79	5.56 ± 3.55	< 0.001
Number of comorbidity	1.31 ± 1.27	1.91 ± 1.44	< 0.001	1.87 ± 1.52	2.52 ± 1.60	< 0.001
K-ADL (score)	7.08 ± 0.58	7.65 ± 1.91	< 0.001	7.09 ± 0.57	7.40 ± 1.40	< 0.001
SGDS-K (score)	2.21 ± 2.33	10.94 ± 2.23	< 0.001	2.87 ± 2.53	11.06 ± 2.23	< 0.001
MMSE-KC (score)	26.21 ± 3.01	23.72 ± 4.46	< 0.001	23.95 ± 4.05	20.90 ± 4.93	< 0.001
Death, n (%)	249 (5.2)	181 (15.3)	< 0.001	144 (2.5)	122 (5.4)	< 0.001
Absolute Handgrip strength (kg)	32.12 ± 7.15	27.30 ± 8.01	< 0.001	19.98 ± 5.12	16.94 ± 5.04	< 0.001
Relative Handgrip strength (kg/BMI)	2.66 ± 0.61	2.32 ± 0.70	< 0.001	1.59 ± 0.44	1.36 ± 0.44	< 0.001
Physical activity (Mets/week)	2122.6 ± 2148.0	1079.2 ± 1242.7	< 0.001	1418.7 ± 1684.8	907.2 ± 1242.7	< 0.001

For continuous variables, values are mean; *BMI* Body mass index, *K-ADL* Korean Activities of Daily Living, *MMSE-KC* Korean version of mini-mental state examination, *SGDS-K* Korean version of 15-item geriatric depression scale. ^a^Men with no depression (*n* = 80) and depression (*n* = 34) and women with no depression (*n* = 156) and depression (n = 73) had no data available. ^b^Men with no depression (*n* = 1246) and depression (*n* = 226) and women with no depression (*n* = 1003) and depression (*n* = 298) had no data available

Table 4 represents the independent and joint effects of low HGS and presence of depression at baseline on the risk of all-cause mortality stratified by gender. In the total sample, individuals with depression only (HR = 1.505, 95% CI = 1.129–2.006, *p* = 0.005) or low HGS only (HR = 1.628, 95% CI = 1.344–1.973, *p* < 0.001) or both exposures (HR = 3.194, 95% CI = 2.635–3.871, p < 0.001) had significantly higher risk of all-cause mortality, compared with individuals with no depression and high HGS (HR = 1). The independent effect of depression only (HR = 1.366, 95% CI = 1.033–1.807, *p* = 0.029) as well as the joint effect of depression plus low HGS (HR = 1.961, 95% CI = 1.409–2.736, *p* < 0.001) remained statistically significant even after adjustments for age, sex, BMI, income, education, alcohol consumption, nutritional risk, K-ADL and MMSE scores, and physical activity.

**Table 4:** Independent and joint effects of handgrip strength and depression on all-cause mortality stratified by sex

Gender	Exposures		Crude HR (95% CI)	P value	Adjusted HR (95% CI)^a^	P value
	Low HGS	Depression				
Total	Negative	Negative	1		1	
	Negative	Positive	1.505 (1.129 ~ 2.006)	0.005	1.366 (1.033 ~ 1.807)	0.029
	Positive	Negative	1.628 (1.344 ~ 1.973)	< 0.001	1.208 (0.782 ~ 1.866)	0.394
	Positive	Positive	3.194 (2.635 ~ 3.871)	< 0.001	1.961 (1.409 ~ 2.736)	< 0.001
Men	Negative	Negative	1		1	
	Negative	Positive	1.682 (1.1.07~2.554)	0.015	1.376 (1.029 ~ 1.841)	0.031
	Positive	Negative	1.786 (1.395 ~ 2.287)	< 0.001	1.120 (0.694 ~ 1.807)	0.643
	Positive	Positive	4.259 (3.308 ~ 5.483)	< 0.001	1.861 (1.306 ~ 2.651)	0.001
Women	Negative	Negative			1	
	Negative	Positive	1.597 (1.070 ~ 2.386)	0.022	0.763 (0.250 ~ 2.325)	0.634
	Positive	Negative	1.394 (1.026 ~ 1.894)	0.034	1.339 (0.440 ~ 4.073)	0.607
	Positive	Positive	2.670 (1.985 ~ 3.591)	< 0.001	2.603 (0.981 ~ 6.908)	0.055

*HR* Hazard ratio, *CI* Confidence interval, *HGS* Handgrip strength. HR^a^ adjusted for age, sex, BMI, income, education, smoking, alcohol consumption, nutritional risk, comorbidities, K-ADL and MMSE scores, and physical activity

Gender-stratified analysis showed that men with depression only (HR = 1.682, 95% CI = 1.107–2.554, *p* = 0.015) or low HGS only (HR = 1.786, 95% CI = 1.395–2.287, *p* < 0.001) or both exposures (HR = 4.259, 95% CI = 3.308–5.483, p < 0.001) had significantly higher risks of all-cause mortality, compared with men with no depression and high HGS (HR = 1). In addition, the independent effect of depression (HR = 1.376, 95% CI = 1.029–1.841, *p* = 0.031) as well as the joint effect of depression plus low HGS (HR = 1.861, 95% CI = 1.306–2.651, *p* = 0.001) remained statistically significant even after adjustments for all the measure covariates.

Women with depression only (HR = 1.597, 95% CI = 1.070–2.386, *p* = 0.022) or low HGS only (HR = 1.394, 95% CI = 1.026–1.894, *p* = 0.034) or both exposures (HR = 2.670, 95% CI = 1.985–3.591, *p* < 0.001) had significantly higher risks of all-cause mortality, compared with women with no depression plus high HGS (HR = 1). However, the independent effect of depression or low HGS was no longer significant, while the joint effect of both depression and low HGS remained significant at borderline (HR = 2.603, 95% CI = 0.981–6.908, *p* = 0.055) when adjustments for all the measured covariates.

## Discussion

In this population-based prospective study, we investigated the independent and joint effects of depression and low HGS at baseline on the risk of 3-year all-cause mortality in Korean older adults. Overall, we found that depression independently contributed to increased all-cause mortality risk in Korean older adults, and the increased all-cause mortality risk was exacerbated by presence of low HGS at baseline. However, our gender-stratified analysis showed that the independent and joint effects of depression and low HGS were only the case for men. In women, the independent effect of depression and low HGS on the risk of all-cause mortality was not observed at statistical significance level, while the joint effect of the exposures remained significant at borderline even after adjustments for all the covariates. Together, the current findings suggest that depression as well as depression plus low HGS are significantly associated with increased all-cause mortality risk in Korean older adults.

With respect to depression, the current findings support and extend those of previous studies reporting gender difference in the association between depression and all-cause mortality in older adults. In a sample of community-living elderly men and women, Schoevers et al. [26] showed that major depressive symptoms was significantly associated with the increased risk of premature death in both men and women. However, the association between minor depressive symptoms and death was found only in men. In a prospective cohort study of 3701 men and women aged > 70 years, Penninx et al. [27] found that newly depressed men were at increased risk of cardiovascular morbidity and mortality, but that women were not. In a large community-based elderly population, Ryan et al. [28] investigated the association between depression and mortality stratified by the use of antidepressants and the severity of depression. In that study, they found that in men, using antidepressants contributed to increased mortality risk according to the severity of depression in a dose-response manner. In women, only the severity of depression in the absence of antidepressants was positively associated with the risk of mortality. On the other hand, by analyzing data obtained from the New Haven cohort (*N* = 2812) of the Established Populations for the Epidemiologic Studies of the Elderly (EPESE) project, Mendes de Leon et al. [29] found that depressed women were at increased risk of coronary heart disease (CHD) mortality and CHD events, but men were not. In a review paper, Sevick and colleagues [30] showed that there is no evidence to support a gender-specific association of depression with major health outcomes in older adults. Together, the gender difference in the independent and joint effects of depression and low HGS on the risk of all-cause mortality observed in the current study should be confirmed in a future study that includes the severity of depressive symptoms, antidepressants, and others as potential confounders.

Several explanations can be given for gender difference in the all-cause mortality risk of depression. First, the gender difference may result from cultural, social, behavioral or adaptive differences between men and women. Women are more likely to report symptoms and distress than men [31], thereby seeking medical treatment. On the other hand, depression is less likely to be recognized and treated in men [32], and therefore the presence of detectable depression in men may signify a more extreme condition [33], accounting for the stronger association with premature death. Second, difference in the nature or intensity of exposure to risk factors or in the vulnerability to the same risk factors may contribute to gender difference in the association between depression and all-cause mortality [28]. Third, genetic and biological factors may some role in the emergence of gender differences in depression and its relation to all-cause mortality [34]. Lastly, in older adults with depression, men are more likely to die and women to be first disabled, which should be tested in a longer follow-up study.

With respect to HGS, the current findings of the study support and extend those of previous studies reporting an inverse association between HGS and the risks of all-cause and cause-specific mortalities in older adults. By analyzing data obtained from a prospective urban-rural epidemiology study involving 139,691 adults aged 35–70 years from 17 countries, Leong et al. [5] showed that HGS was inversely and significantly associated with all-cause mortality (HR = 1.16 and 95% CI = 1.13–1.20), cardiovascular mortality (HR = 1.17 and 95% CI = 1.11–1.24), non-cardiovascular mortality (HR = 1.17 and 95% CI = 1.12–1.21), myocardial infarction (HR = 1.07 and 95% CI = 1.02–1.11) and stroke (HR = 1.09 and 95% CI = 1.05–1.15) during 4 years of follow-up. In a population-based longitudinal cohort study involving 6850 men (42%) and women (58%) aged 50–80 years, Strand et al. [35] showed that weak HGS at baseline was significantly and positively associated with all-cause and cause-specific mortalities during 17 years of follow-up. In a systematic review, Bohannon [36] also showed that low HGS was consistently and significantly associated with premature mortality, disability, and other health-related complications in middle-aged and older adults. In the cooperative health research involving 1066 individuals aged 76 ± 11 years, Arvandi et al. [37] showed that low HGS was significantly associated with increased all-cause mortality risk during > 3 years of follow-up, and women with low HGS had higher all-cause mortality risk than men with low HGS.

Several explanations can be given for the association between low HGS and all-cause mortality risk. Firstly, depression is associated with low HGS, together contributing to increased all-cause mortality risk additively. Secondly, low HGS is significantly associated with increased morbidities of chronic diseases, thereby contributing to increased all-cause mortality risk additively. Thirdly, low HGS may reflect frailty since it is a key component of its phenotype. Frail older adults are less likely to get a chance of medication and/or medical treatments [38] when they are necessary, contributing to increased risk of dying prematurely from all causes. Lastly, low HGS may be the end stage of a cascade of events from inflammatory processes to coagulative dysregulation to a range of alterations in hormones and peptides and thereby a threshold state characterized by increasing inability to adequately address physiological demands or a disruption of homeostatic mechanisms.

Taken together, those findings showed that depression and low HGS contributed to increased all-cause mortality in an addictive manner in older men but not in older women, implying a gender difference in the relationship of depression and low HGS with the risk of premature death from all causes in Korean geriatric population. Yet, the mechanism(s) underlying the gender difference in the impacts of the two exposures on the risk of all-cause mortality remains to be elucidated.

The present study have some strengths. First, study participants were a representative sample of Korean older populations, as described elsewhere [21, 22]. Second, the mortality data were gathered from a reliable register. Third, many covariates as possible were assessed in order to obtain a more reliable and reproducible association between the exposures and mortality. However, some limitations of the study must be also acknowledged. First, any causal inference regarding the pathologic mechanism(s) linking low HGS and depression to all-cause mortality cannot be possible due to the cross-sectional nature of the present study. Second, biomarkers including inflammatory cytokines and hormones associated with loss of muscle mass and/or depression were not available in this study. Third, depression differently modulates the association between low HGS and all-cause mortality, especially among young-old adults [39].

## Conclusion

In summary, low HGS and depression are important public health problems due to their devastating consequences. In this study, we found that depression and low HGS were significantly associated with increased all-cause mortality risk in Korean older adults, suggesting that a multidimensional effort to deal with physical dysfunction and mental health is likely to be more relevant to mortality in Korean geriatric population In men, both independent and joint effects of depression and low HGS were significantly associated with the increased risk of all-cause mortality independent of all the covariates. Likewise, both depression and low HGS were also significantly and synergistically associated with increased all-cause mortality risk in women. Yet, the joint effect of the exposures only remained significant at borderline when adjusted for all the covariates. Considering the cross-sectional nature of the study, however, a further study will be necessary to investigate how the covariates influence the independent and joint effects of low HGS and depression on all-cause mortality in a cause- and-effect manner in Korean older adults.

## Abbreviations

CHD: Coronary heart disease
CVD: Cardiovascular disease
EPESE: Established Populations for the Epidemiologic Studies of the Elderly
HGS: Handgrip strength
HR: Hazard ratio
IPAQ: International Physical Activity Questionnaire
K-ADL: Korean activities of daily living scale
LLD: Late life depression
LPOPS: Living Profiles of Older People Survey
MMSE-KC: Korean version of the mini-mental state examination
SGDS-K: Korean version of the short form of the geriatric depression scale
